# Supplementary material for: Complexome profiling of the Chlamydomonas psb28 mutant reveals TEF5 as an early PSII assembly factor
Source: Plant Cell. 2025 Mar 17;37(6):koaf055. doi: 10.1093/plcell/koaf055 (PMC12164590; doi:10.1093/plcell/koaf055)
Supplement: koaf055_Supplementary_Data [file koaf055_supplementary_data.zip › psb28_interactive.zip!/plots/intensity/qualityPlots/peptides/ERD2C_Cre06.g309350.t1.2.html]

### Cre06.g309350.t1.2

Trivial name: ERD2C  
SeqCoverage [median]: 4.40  
Mass: 28.46 kDa  
  
 

| Sequence | MissedCleavage | Mass [Da] | wtIntensity | muIntensity | Score |
| --- | --- | --- | --- | --- | --- |
| FKFEDQEANRK | 2 | 1410.69 | 1436.96 | 9124.09 | 117.25 |
|  |  |  | 1437.0 | 9124.1 |  |
